# Peroxiredoxin 6 Down-Regulation Induces Metabolic Remodeling and Cell Cycle Arrest in HepG2 Cells

**DOI:** 10.3390/antiox8110505

**Published:** 2019-10-23

**Authors:** María José López Grueso, Rosa María Tarradas Valero, Beatriz Carmona-Hidalgo, Daniel José Lagal Ruiz, José Peinado, Brian McDonagh, Raquel Requejo Aguilar, José Antonio Bárcena Ruiz, Carmen Alicia Padilla Peña

**Affiliations:** 1 Department of Biochemistry and Molecular Biology, University of Córdoba, 14074 Córdoba, Spain; q02logrm@uco.es (M.J.L.G.); rosa.tarradas.valero@gmail.com (R.M.T.V.); cahibeatriz@gmail.com (B.C.-H.); b32larud@uco.es (D.J.L.R.); bb1pepej@uco.es (J.P.); bb2reagr@uco.es (R.R.A.); bb1papec@uco.es (C.A.P.P.); 2 Maimónides Biomedical Research Institute of Córdoba (IMIBIC), 14004 Córdoba, Spain; 3 Department of Physiology, School of Medicine, NUI Galway, H91 TK33 Galway, Ireland; BRIAN.MCDONAGH@nuigalway.ie

**Keywords:** Peroxiredoxin, thiol redox regulation, redox proteome, redox homeostasis, lipid metabolism, cell cycle

## Abstract

Peroxiredoxin 6 (Prdx6) is the only member of 1-Cys subfamily of peroxiredoxins in human cells. It is the only Prdx acting on phospholipid hydroperoxides possessing two additional sites with phospholipase A2 (PLA2) and lysophosphatidylcholine-acyl transferase (LPCAT) activities. There are contrasting reports on the roles and mechanisms of multifunctional Prdx6 in several pathologies and on its sensitivity to, and influence on, the redox environment. We have down-regulated Prdx6 with specific siRNA in hepatoblastoma HepG2 cells to study its role in cell proliferation, redox homeostasis, and metabolic programming. Cell proliferation and cell number decreased while cell volume increased; import of glucose and nucleotide biosynthesis also diminished while polyamines, phospholipids, and most glycolipids increased. A proteomic quantitative analysis suggested changes in membrane arrangement and vesicle trafficking as well as redox changes in enzymes of carbon and glutathione metabolism, pentose-phosphate pathway, citrate cycle, fatty acid metabolism, biosynthesis of aminoacids, and Glycolysis/Gluconeogenesis. Specific redox changes in Hexokinase-2 (HK2), Prdx6, intracellular chloride ion channel-1 (CLIC1), PEP-carboxykinase-2 (PCK2), and 3-phosphoglycerate dehydrogenase (PHGDH) are compatible with the metabolic remodeling toward a predominant gluconeogenic flow from aminoacids with diversion at 3-phospohglycerate toward serine and other biosynthetic pathways thereon and with cell cycle arrest at G1/S transition.

## 1. Introduction

Peroxiredoxins (Prdx) are ubiquitous, abundant, and highly conserved enzymes whose main function is to catalyze the reduction of peroxides, taking part in the antioxidant battle of cells against ROS. They all possess a catalytic or “peroxidatic” Cys residue that acts as a hydrogen donor to the peroxide substrate and have been classified in six subfamilies, of which three are present in mammalian cells [1]. Human Prdx1 to Prdx4 belong to the “typical 2-Cys type” and Prdx5 belongs to the “atypical 2-Cys” subfamily. They have a second “resolving” Cys that plays a critical role in the catalytic cycle and is then reduced by thioredoxin (Trx). Human Prdx6 belongs to the “1-Cys” subfamily, which, unlike the other members of the Prdx family, does not possess a “resolving Cys” and may substitute Trx by glutathione in the final step of the catalytic cycle [2,3,4,5]. Mouse and human Prdx6 have an additional non-conserved Cys residue (Cys91 in human) with a so far unknown function.

Several members of the Prdx family are moonlighting proteins that have acquired additional functions during evolution. For instance, some Prdx of the 2-Cys subfamily can form oligomers with chaperone activity when overoxidized [6] or can act as sensors and transmitters of H_2_O_2_ signaling [7]. Prdx6 is peculiar and stands out from the rest of Prdx for its multiple functions. It is the only Prdx acting on phospholipid hydroperoxides and has two additional catalytic sites: one is responsible for acidic calcium-independent phospholipase A2 activity (PLA2) [8] and the other displays lysophosphatidylcholine acyl transferase (LPCAT) activity [9]. It has been proposed that these outstanding properties confer on Prdx6 the capacity to fulfill important functions associated with phospholipid turnover and membrane repair [10]. Posttranslational modifications and changes in subcellular localization regulate the actions of Prdx6. Phosphorylation of Thr177 by MAPK [11] and overoxidation of peroxidatic Cys47 [12,13] stimulate the PLA2 activity. Prdx6 is present in the cytoplasm but can translocate to the plasma and mitochondrial membranes and can also associate with lysosomal-type organelles [14,15]. Association of Prdx6 with plasma membrane contributes to NADPH (reduced nicotinamide adenine dinucleotide phosphate) oxidase (NOX1, NOX2) activity [16] and translocation to the mitochondria takes part in the initiation of mitophagy [15].

Its multiple functionality makes Prdx6 an important player in cell physiology and pathology [17,18] and both peroxidase and PLA2 activities have been associated with tumor progression [19] and dopaminergic neuron degeneration [20]. However, contrasting findings have been reported concerning its roles in cancer. Down-regulation of Prdx6 with specific siRNA decreased proliferation and induced apoptosis in canine haemangiosarcoma cells [21], whereas overexpression of Prdx6 attenuated apoptosis in ovarian cancer cells [22] and promoted growth of lung tumors in mice through activation of JAK2/STAT3 signaling [23]. It has also been reported that Prdx6 either enhance tumor multiplicity or reduce tumor number in mice skin cells, depending on the stage of tumor development [24]. Both peroxidase and PLA2 activities of Prdx6 were required for tumor development in xenografted mice lung in a process dependent on arachidonic acid (AA) release [25]. AA production and NOX2 activation was also involved in stimulation of metastasis by Prdx6 PLA2 activity in several cancer models [25,26], although in another study, NOX2 activation was shown to proceed through Prdx6 mediated lysophosphatidic acid (LPA) receptor activation [27]. Prdx6 has also been related to pathogenesis of cardiometabolic diseases [17] and inflammatory diseases with contrasting roles of peroxidase and PLA2 activities [18].

In a recent study, we have found that weakening the cell’s antioxidant potential by experimental down-regulation of glutaredoxin 1 (Grx1) in HepG2 cells affects the phospholipid metabolism and the redox state of Prdx6 “extra residue” Cys91 [28]. In view of the above-outlined background of contrasting reports on the roles and mechanisms of multifunctional Prdx6 in several pathologies and its sensitivity to the redox environment, we have undertaken a project based on down-regulating Prdx6 with specific siRNA in hepatoblastoma HepG2 cells to study the role of Prdx6 in cell proliferation, redox homeostasis and metabolic programming. Silencing with specific siRNA provides a good experimental approach to mimic physiological down-regulation events that may occur as part of a cell response to various stimuli. We have found that the cells respond to Prdx6 silencing by adapting carbon and lipid metabolism and by modulating signaling pathways to stop cell cycle progression at the G1/S phase transition.

## 2. Materials and Methods

### 2.1. Materials and Reagents

All reagents were of analytical grade and were purchased from Sigma (St. Louis, Missouri, USA) unless otherwise specified. HepG2 cell line used in this work was obtained from the ATCC LGC Standards Company (Teddington, UK). Cell culture dishes and flasks were from TPP (Switzerland). The specific small interfering RNA for PRDX6 (siRNAPrdx6) and non-target (NT) were from Dharmacon (Lafayette, CO, USA) and ECL was from GE Healthcare (Wauwatosa, Wisconsin, USA). Antibodies against PRDX6 were from Abcam (Cambridge, UK). Antibodies against CD95 were from Santa Cruz Biotechnology, (Dallas, TX, USA). Antibodies against Actin were from Sigma.

### 2.2. Cell Growth Conditions, Proliferation and Cell Viability

Cells were grown in EMEM Medium (Eagle Minimum Essential Medium), pH 7.4, supplemented with 10% fetal bovine serum, 2.2 g/L NaHCO3, 1 mM sodium pyruvate, 100 U/L penicillin, 100 µg/mL streptomycin, and 0.25 µg/mL amphotericin in 5% CO2 atmosphere at 37 °C. Cell proliferation was analyzed using a colorimetric ELISA (Roche Applied Science, Penzberg, Germany). 20,000 cells/cm^2^ were cultured in 96-well multiplates at 37 °C and 5% CO_2_. 72h after siRNAPrx6 addition HepG2 cells were incubated with 10 µM BrdU labeling solution for 6h at 37 °C. Then cells were fixed with 200 µL of FixDenat solution for 30 min at room temperature. From this point, the incubations were done at room temperature. After this time, fixed cells were incubated with 100 µL of anti-BrdU-POD solution with a previous dilution of 1: 100 for 90 min, and washed with PBS. Finally, fixed cells were incubated with 100 µL of substrate solution for 30 min. Total number of cells and cell viability in a HepG2 cell suspension were quantified using the trypan blue dye exclusion method in 90 mm dishes.

### 2.3. Silencing of Prdx6

Human Prdx6 was down-regulated in HepG2 cells using a specific pool of four small interfering RNA (Ref. L-019173-00-0005) according to the manufacturer’s recommendations (Dharmacon, GE Healthcare, Life Science). 20,000 cells/cm^2^ were cultured in dishes and treated with 25 nmol of siRNA mixed with the transfection reagent DharmaFECT-1 preincubated with culture medium (antibiotic and serum free) for 20 min at RT, in a 1:2 proportion and the interference solution was kept for 72 h to obtain the maximum inhibition of ≈60%. Non-targeting (NT) negative controls were run in parallel. Silencing of Prxd6 was always checked by Western blot.

### 2.4. Measurement of Cell Death and Area of Cell Nuclei

Apoptosis in HepG2 cells was analyzed through Annexin V-FITC (Canvax, Córdoba, Spain). Annexin-V binds to phosphatidylserine (PS) residues which are on the outer plasma membrane of apoptotic cells. Annexin-V is labeled with the fluorophore FITC to visualize apoptotic cells by fluorescent microscopy. 20,000 cells/cm^2^ were cultured on a coverslip introduced in each well of a 24-well plate at 37 °C and 5% CO_2_. 2.5 µL of Annexin-V-FITC was added after siRNAPrdx6 following the instructions of the manufacturer. Then, cells were fixed in methanol and permeabilized with 0.2% Triton-X100 solution in PBS. Cells nuclei were stained using DAPI. Fluorescence was measured with a fluorescence microscope (Olympus BX43) using standard fluorescein filter set to view Annexin-V-FITC fluorescence at 520 nm and view blue DAPI at 460 nm. The area of cell nuclei was measured on the DAPI pictures using the open source software “ImageJ” [29].

### 2.5. Measurement of Enzymatic Activities, Glucose and Protein

HepG2 PLA2 activity was assayed using the Red/Green BODIPY based EnzChek Phospholipase A2 Assay (Invitrogen) (pH 7.0, in the absence of calcium) in 96-well plates [30]. Cells were sonicated (4 × 5 s) in PLA2 buffer (50 mM Tris-HCl, 1 mM EGTA, pH 7.0) supplemented with protease and phosphatase inhibitors, and the supernatant collected by centrifugation at 15,000× *g* for 15 min was adjusted to ≈50 µg protein and preincubated for 10 min at 37 °C in the absence or presence of MJ33 (10 µM) [31]. At 60 min, samples were analyzed in a Microplate reader (Varioskan, Thermo Scientific) at an excitation of 460 nm and emissions of 515 nm. Background was subtracted and the ±MJ33 differential 515 nm emission were recorded. Caspase-3, caspase-8, and caspase-9 associated activities were determined using the corresponding fluorescence peptide-based substrates (100 µM) in the reaction mixture (50 mM HEPES pH7.5, 100 mM NaCl, 10% sucrose, 0.1% Chaps, 1 mM EDTA and 5 mM DTT). The substrates used were Ac-DEVD-AFC, Ac-LETD-AFC, and Ac-LEHD- AFC for caspase-3, -8, and -9, respectively. The fluorescence due to the reaction product was recorded with a GENIos Microplate Reader (TECAN) set at 400 nm excitation and 505 nm emission. Glucose concentration in the culture medium was determined by a standard enzymatic colorimetric assay at 505 nm (LabKit, Chemelex, S.A., Spain). Protein concentration in the samples was determined by the Bradford method (Bio-Rad) using BSA as the standard.

### 2.6. Proteomics

#### 2.6.1. Sample Preparation and Mass Spectrometry

The experiments were routinely carried out at 20,000 cells/cm2 and a modification of the methodological strategy described previously [28] was used. Reduced cysteines were blocked with “light” Iodoacetamide (IAM) while reversibly oxidized Cys were labeled with “heavy” IAM (Iodoacetamide-13C2, 2d2) incorporating ∆mass of 4 Da. Cell extracts were obtained with a lysis solution (50 mMTris-HCl pH 8.5, 5 mM IAM light, 0.5% CHAPS, 1 mM PMSF); samples were centrifuged at 15,000g for 5 min at 4 °C. 100 µg of protein were diluted up to 80 µL with 50 mM Tris-HCl pH 8.5 and incubated with 4 µL of 160 mM DTT at 37 °C for 30 min to reduce the reversibly oxidized cysteines. Subsequently, cysteines were alkylated adding 6 µL of 240 mM IAM heavy and incubated at room temperature for 30 min in darkness. Finally, the excess of IAM and CHAPS was removed using Zeba spin desalting columns (Thermo Scientific) equilibrated with 25 mM ammonium bicarbonate pH 7.0. The level of silencing was checked at this point by SDS-PAGE.

Proteolytic digestion and proteomic analyses were performed as described previously [28]. The spectrometer used was the Thermo Orbitrap Fusion (Q-OT-qIT, Thermo Scientific) equipped with a nano-UHLC Ultimate 3000 (Dionex-Thermo Scientifics).

#### 2.6.2. Label-Free MS Protein and Redox Quantification

The “label-free” quantification was performed using the MaxQuant (v1.5.7.0) free software [32] configuring “light” IAM and “heavy” IAM and methionine oxidation as variable modifications. No imputation of missing values was implemented. Finally, only the conditions with three or more values per identification were considered and analyzed using a Student’s T-test. The criteria for considering a differentially expressed protein were that it was identified and quantified using at least two unique peptides and it showed a fold change of at least 1.5 and a *p* ≤ 0.05 value. MS2 spectra were searched with SEQUEST engine against a database of Uniprot_Human_Nov2014 (www.uniprot.org). The Cys-containing peptides labeled with light and heavy IAM modifications independently were quantified using the open software Skyline [33]. The individual reduced/oxidized ratio for specific Cys residues was calculated in each sample in order to obtain the redox proteome. The intensities of misscleavaged peptides were taken into account for the calculations.

### 2.7. Metabolomics

The metabolomic study is part of a larger study and the analyses were performed at Metabolon, NC USA (www.metabolon.com). The samples from four different experiments were prepared following the company specific guidelines as described before [28]. Briefly, dry cell pellets were immediately frozen in liquid nitrogen and stored at −80 °C until shipment. Samples were prepared using the automated MicroLab STAR^®^ system from Hamilton Company. To remove protein, dissociate small molecules bound to protein or trapped in the precipitated protein matrix, and to recover chemically diverse metabolites, proteins were precipitated with methanol under vigorous shaking for 2 min (Glen Mills GenoGrinder 2000) followed by centrifugation. The resulting extract was divided into five fractions: two for analysis by two separate reverse phase (RP)/UPLC-MS/MS methods with positive ion mode electrospray ionization (ESI), one for analysis by RP/UPLC-MS/MS with negative ion mode ESI, one for analysis by HILIC/UPLC-MS/MS with negative ion mode ESI, and one sample was reserved for backup. Extraction of raw data, peak identification and quantification, and QC were performed using Metabolon’s hardware and software.

### 2.8. SDS-PAGE and Western Blotting

SDS-PAGE was performed in 12% Criterium XT Precast Gels (Bio-Rad) for detection of specific proteins (Prdx6, CD95, Actin). After electrophoresis, proteins were transferred to a nitrocellulose membrane in a semi-dry electrophoretic transfer system (Bio-Rad) for 40 min at 400 mA constant. Transfer and protein load were checked by staining with Ponceau reagent. The membranes were incubated overnight at 4 °C with the corresponding dilutions of primary antibodies: 1:500 CD95, 1:2000 Prdx6, and 1:4000 Actin. They were then washed with TBS-T and incubated with the corresponding secondary antibodies conjugated to peroxidase (anti-rabbit, anti-goat, or anti-mouse) used at 1:8000 dilution. The chemiluminescent signal induced by ECL reagent (GE Healthcare) was detected in a ChemiDoc image analyzer (Bio-Rad) and quantified by densitometry with Quantity-One 1-D analysis software (Bio-Rad) using Actin as reference for loading normalization.

### 2.9. Statistics

Where appropriate, results are expressed as mean ± SD of at least three independent experiments and Student *t*-test used for significance. Large data sets were analyzed using Storey and Tibshrani method [34] comparing non-target control vs. siRNA Prdx6 treated. The threshold for statistically significant differences was set at *p*-value adjust ≤0.05; *q*-values are also included as an additional reference.

## 3. Results and Discussion

### 3.1. Prdx6 down-Regulation Reduced Cell Proliferation

The peroxidatic Cys47 in Prdx6 is predominantly (>60%) in a reversibly oxidized state in HepG2 cells (see data in Supplementary File S2) but its overoxidation would decrease the peroxidase activity and stimulate the PLA2 activity [13].

**Figure 1:**
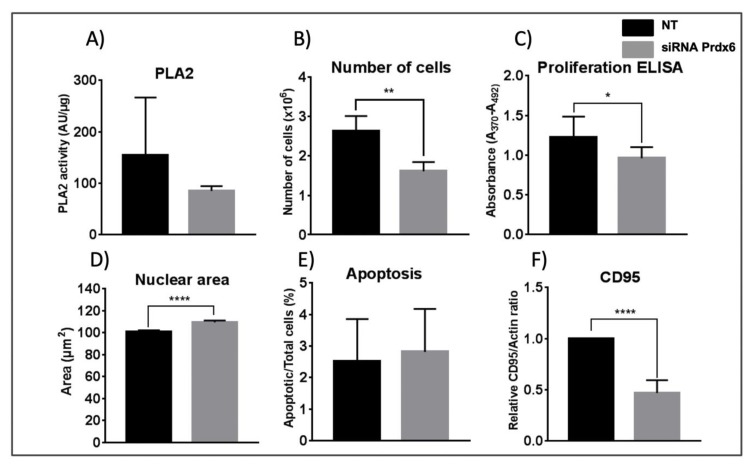
Effect of Prdx6 silencing on PLA2 activity, cell viability, nuclear area, apoptosis, and CD95 level. (**A**) Prdx6-dependent PLA2 activity was determined, as described in Materials and Methods, in cell lysates of HepG2 cells treated with siRNA-Prdx6 or with non-target siRNA (NT) as indicated. Activity is expressed as arbitrary fluorescence units normalized for protein content. Data are the mean of 5 independent biological replicates. (**B**) The number of cells determined as indicated in M&M. (**C**) Proliferation in terms of cells entering the S phase was determined by the degree of BrdU incorporation to replicating DNA and expressed in absorbance units. (**D**) The nuclear area of cells was determined as indicated in M&M. >500 cells were measured, from 6 different slides, in each sample. The area is given in arbitrary units. (**E**) Apoptosis was determined by the Annexin V fluorescence method; the number of positive cells relative to the total number of cells in percentage is represented. (**F**) CD95 levels were determined by western blotting with specific antibodies and band density was normalized to the signal for actin. Parameters in (B), (C), (E) and (F) were determined in three independent experiments; error bars show SD; statistical significance was assessed by Student’s *T*-test and is shown with a number of asterisks inversely proportional to the *p*-value (**** < 0.0001, ** < 0.01, * < 0.05).

It was not possible to measure the Prdx6-specific peroxidase activity in cell lysates, but its phospholipase A2 activity (PLA2) was determined using a standard fluorimetric assay in the presence of MJ33, a Prdx6 specific PLA2 inhibitor, as described in Materials and Methods. The assay gives a high variability and, although the resulting data from five different experiments were not statistically significant, a trend was observed with lower activity in siRNA-Prdx6 treated cells (Figure 1A) in parallel with decreased levels of Prdx6 protein by ≈60%, as determined by Western blot and confirmed by mass spectrometry quantitative analysis (See data in Supplementary File S1).

The number of cells and incorporation of BrdU, indicative of the number of cells in the S phase or proliferation, both decreased after 72 h treatment with siRNA-Prdx6 (Figure 1B,C). Cell viability did not change in either condition (data not shown), but the size of the cells augmented as evidenced by a significant increase in nuclear size (Figure 1D). The nuclear size is an important parameter governing entry into the cell cycle. These results suggest cell cycle blockade induced by Prdx6 down-regulation.

### 3.2. Prdx6 Silencing Interferes with Apoptotic Signaling from CD95 but does not Induce Apoptosis in HepG2 Cells

To check whether the anti-proliferative action of Prdx6 down-regulation could be due to activation of cell death mechanisms, we analyzed several apoptotic parameters. Prdx6 silencing did not affect apoptosis significantly in HepG2 cells as shown by Annexin V fluorescence (Figure 1 E), but did induce a marked decrease in CD95 protein levels (Figure 1F). This effect could not be associated to caspase modulation, since the levels and activities of caspases 3, 8 and 9 did not change significantly, although caspase 3 displayed unusually high variability (data not shown). It is well established that activation of CD95 receptor mediates the induction of apoptosis mostly in the context of immune response. CD95 is ubiquitously expressed in all types of cells, including cancer cells, but its expression in cancer cells implies that they are themselves resistant to CD95-mediated apoptosis [35]. It is now widely accepted that CD95 has multiple non-apoptotic functions and that stimulation of CD95 in cancer cells can turn this death receptor into tumor promoting receptor [35] through diverse tumorigenic signaling pathways. In lung cancer cells, activation of CD95 induced the production of proinflammatory factor PGE2 through the p38 pathway, and promoted cell growth that was reduced by administration of the cyclooxygenase-2 inhibitor [36]. Interestingly, accelerated entry into the cell cycle S-phase by CD95 stimulation was demonstrated in pancreatic ductal adenocarcinoma cells in vitro in a process not dependent on the DISC (death-inducing signaling complex) formation but mediated by recruitment of the adapter protein Sck (SH2 domain protein C2) [37].

Altogether, these results show that the response of HepG2 cells to Prdx6 silencing is rather complex, with a mixture of apparently contradictory outcomes like CD95 down-regulation but no changes in caspases and cell swelling. It would be worth determining whether the antiproliferative action could be due to the blockade of entry into cell cycle S-phase by down-regulation of CD95 and the possible involvement of signaling events dependent on the PLA2 activity of Prdx6. These aspects will be discussed below in a wider context including changes at metabolomic, proteomic, and redox levels.

### 3.3. Metabolic Remodeling after Prdx6 Silencing

Metabolic remodeling and protein posttranslational modification (PTM) are the first levels of cellular response to a stimulus before a stable response is set up at the transcriptional level. We carried out a metabolomic analysis (Supplementary File S3) to determine the possible effect of Prdx6 down regulation in the metabolic profile of HepG2 cells.

#### 3.3.1. Lipids

Lipid metabolism plays a critical role in cell survival and proliferation since these varied molecules are indispensable for the structural, signaling, and energy needs of every cell. Phospholipids are synthesized during entry in the cell cycle but the synthesis pauses for cell division, so that the G1 and S phases are characterized by doubling of phospholipid content [38].

**Figure 2:**
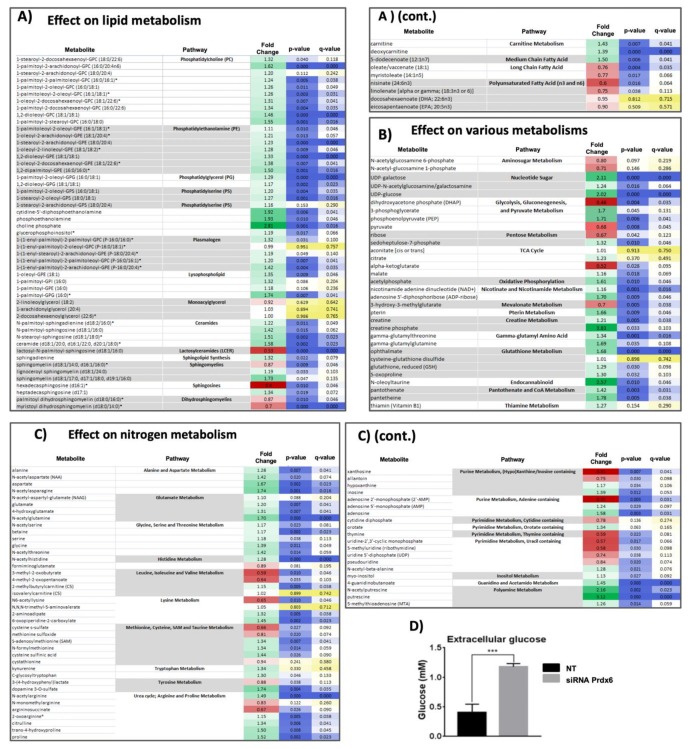
Summary of significant metabolomic changes. A metabolomic analysis was carried out as described in Materials and Methods. The fold change (up-regulation, green; down-regulation, red) of each metabolite level on Prdx6 silencing in HepG2 cells is shown in the first column; the second and third columns show the *p*-values and *q*-values, respectively, for statistical significance; *p* and *q*-values ≤0.05 are in blue with the color intensity inversely proportional to *p*- or *q*-value. The results have been clustered into three figures and into pathways therein: (**A**) Metabolites of lipid metabolism; (**B**) metabolites of other pathways and (**C**) metabolites of aminoacids and nucleotides metabolism. (**D**) The concentration fold change of extracellular glucose determined by standard colorimetric methods in three independent experiments (*** *p* < 0.001).

Increased levels of all kinds of phospholipids, lysophospholipids, plasmalogens and ceramides, with the exception of lactosylceramide, was a marked trend in siRNA-Prdx6 treated cells. A parallel increase in UDP-glucose and UDP-galactose levels (Figure 2B) is consistent with the enhanced rate of glycolipids synthesis. CDP-ethanolamine, P-choline and P-ethanolamine of the Kennedy pathway were particularly elevated. Augmented levels of phosphocholine have been observed in a wide variety of human cancers, caused in part by the growth factor-activated Ras and phosphatidylinositol 3-kinase (PI3K) signaling cascades that stimulate the initial enzyme of the choline branch of the pathway [39]. It would be valuable to know if the elevated levels of these phospholipid precursors caused by Prdx6 silencing involve this signaling pathway. Increased phospholipid content could contribute to the observed increase in the cell volume described above (Figure 1D).

Long chain monoacylglycerols (MAG) were an exception to this trend of increased lipid content Some MAG are known as endocannabinoids, like 2-arachidonyl-glycerol and it has been recognized that they have important signaling functions [40]. MAG can form through the action of PLA2 on phosphatidic acid (PA) while their degradation is carried out by membrane bound MAG hydrolases of which two types have been described, the ubiquitously expressed serine hydrolase α/β-hydrolase domain 6 (ABHD6) and classical MAG lipase, a housekeeping enzyme. ABHD6 is expressed at high levels in certain tumors and its pharmacological or genetic suppression, which leads to an elevation in 1- and 2-MAG content in different tissues, appears to have multiple health benefits, likely mediated via MAG signaling [40]. Only three members of this family of lipases were detected in the proteomic analysis (ABHD10, ABHD12, and ABHD14B; see data in Supplementary File S1) and none of them showed significant quantitative changes on Prdx6 silencing, stressing the possible contribution of Prdx6 PLA2 activity down-regulation to unchanged MAG levels.

Long chain fatty acids decreased in siRNA-Prdx6 treated cells while the levels of carnitine followed a reciprocal trend (Figure 2A cont.). The accumulation of carnitine is indicative of reduced entry of long chain fatty acids into the mitochondria for ß-oxidation. A natural variant C304➝W (VAR_020548; dbSNP: rs80356776) of carnitine O-palmitoyltransferase 1 (CPT1A) with pathological consequences produces an unstable, inactive form of the enzyme [41] stressing the importance of Cys residues for optimal import of long chain fatty acids into the mitochondria. Redox changes observed in CPT1A (See Supplementary File S2) could be relevant to these trends in long chain fatty acid metabolism. Our results show that CPT1A-Cys96 was oxidized (0.3 red/ox ratio fold change; *p* = 0.005) on siRNA-Prdx6 silencing. Reversible oxidation of CPT1A-Cys96 would slow down fatty acid ß-oxidation in favor of increased biosynthesis and incorporation into phospholipids in siRNA-Prdx6 treated cells.

There could also be a relationship between the levels of unsaturated long chain fatty acids and PLA2 activity of Prdx6. Early characterization of Prdx6 PLA2 activity showed high activity with arachidonic acid (AA) containing phospholipids [42,43]. A proapoptotic role of Prdx6 via induction of AA release by its PLA2 activity has been postulated in human bronchial epithelial cells [44] but another study found that AA production by PLA2 activity of Prdx6 induced cell proliferation through activation of Src family kinases in human melanoma cells [26]. These contradictory results reveal the complexity of Prdx6 action mechanisms and highlight the importance of lipid signaling in cancer.

#### 3.3.2. Nitrogen Metabolism

A generalized increase in aminoacids, with few exceptions, was observed that could be a consequence of increased biosynthesis and/or protein degradation. A marked similar trend was observed for polyamines, a sign of the cells being prepared for proliferation since elevated polyamine levels, are necessary for transformation and tumor progression [45]. This increment could also contribute to the above-mentioned increase in cell volume (Figure 1D). It would be worth checking whether these changes are due to alteration in the PI3/Akt/mTOR pathway.

There was an overall decrease in metabolites of the purine and pyrimidine metabolism subfamilies, with the exception of adenosine, inosine, and hypoxanthine upon Prdx6 silencing. These results are indicative of diminished nucleotide biosynthetic activity and activation of adenine catabolism, although urate was not detected.

#### 3.3.3. Other Metabolites

Several metabolites of the glutathione metabolism subfamily were elevated in siRNA-Prdx6 treated cells likely as a consequence of glutathione demand in the face of diminished antioxidant defenses. A conspicuous increase of the Glutamate and Serine, Threonine, and Glycine pathways was also detected at metabolome level reinforcing the idea of metabolic pathways converging on glutathione synthesis.

**Scheme 1:**
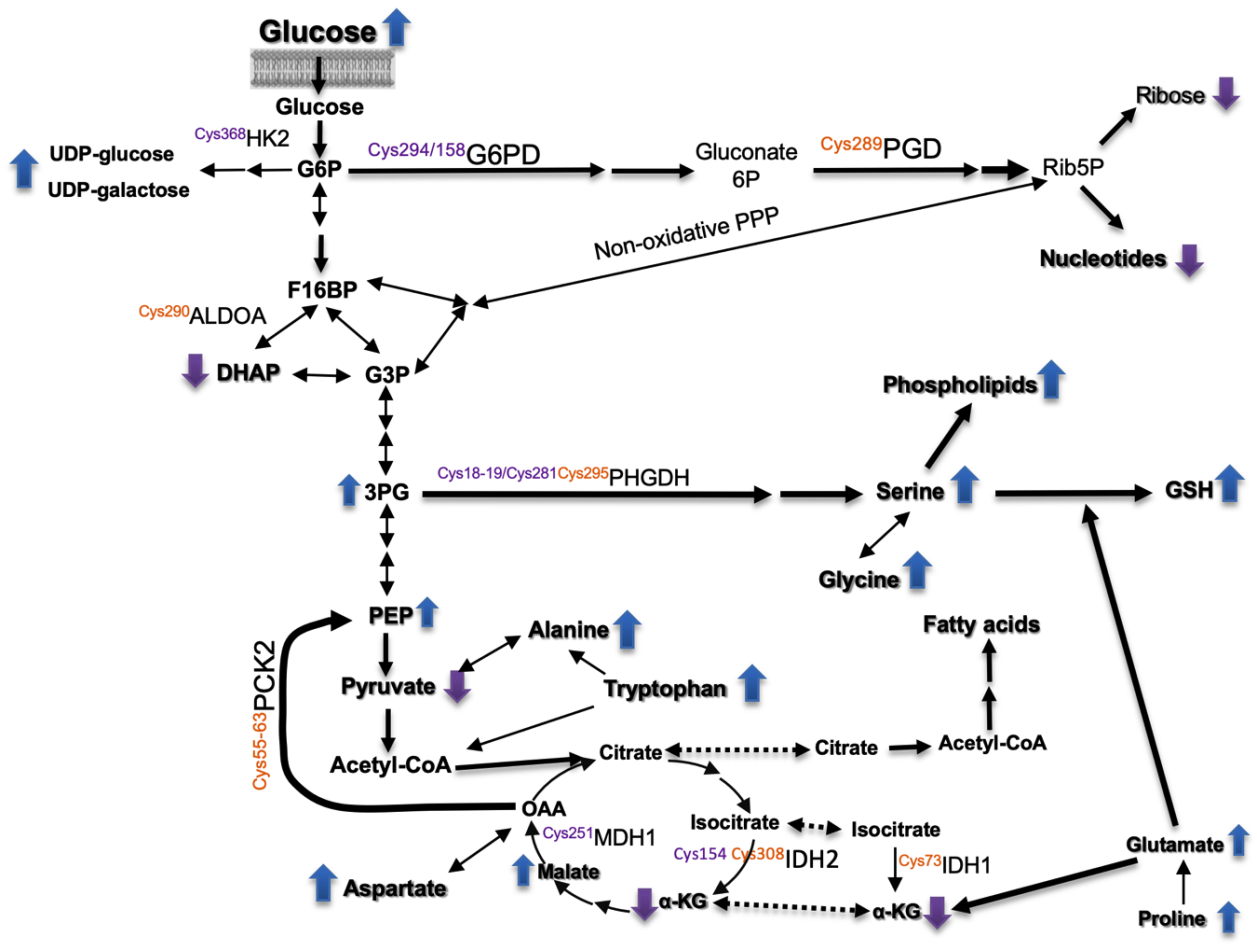
Overview of metabolic pathways affected by Prdx6 down-regulation. Relative levels of metabolites and thiol redox changes in enzymes in siRNA-Prdx6 treated HepG2 cells. Up and down arrows indicate increased or decreased levels, respectively. Mapped redox sensitive Cys showing a differential redox state (oxidative purple; reductive, orange). See main text for detailed explanation.

Regarding carbon metabolism (Figure 2B), treatment of HepG2 cells with siRNA-Prdx6 lowered dihydroxyacetone-phosphate (DHAP), pyruvate, ribose, and 2-oxoglutarate (αKG) levels whereas extracellular glucose (Figure 2D), phosphoenol-pyruvate (PEP), 3-phosphoglycerate (3-PG), sedoheptulose-7-phosphate (Shu7P), malate, aspartate, and serine increased. These changes are compatible with reduced import of glucose and diminished flow through the upper glycolytic pathway, the non-oxidative part of the Pentose-phosphate pathway (PPP) and the nucleotide biosynthetic pathway. In the lower part of the pathway a predominant gluconeogenic flow from aminoacids with diversion at 3-PG toward serine and other biosynthetic pathways thereon would be taking place (Scheme 1).

Changes detected in the redox proteome could be relevant to these metabolic set up, since glucose-6-phosphate and 6-phosphogluconate dehydrogenases (G6PD and PGD), aldolase (ALDOA), hexokinase (HK2), malate dehydrogenase (MDH1), PEP carboxykinase (PCK2), and 3-phosphoglycerate dehydrogenase (PHGDH) showed redox changes at specific Cys residues that could affect their catalytic performance (highlighted in Scheme 1 and commented below).

### 3.4. Changes at Proteome Level

The extent of down-regulation of Prdx6 under our experimental conditions was not incisive enough to provoke extensive changes at global proteome level through transcriptional regulation, but had a marked effect at posttranslational level as observed by changes in the redox proteome.

#### 3.4.1. Global Proteome

As a proof of concept, the quantitative global proteomic analysis (Supplementary File S1) detected 0.5-fold Prdx6 down-regulation in silenced cells. The number of differentially expressed proteins after siRNA-Prdx6 treatment was rather low. However, a group of differential proteins on Prdx6 silencing were significantly enriched in endocytosis (GO:0030100 term and hsa04144 KEGG pathway): the ADP-ribosylating factor GTPase-activating protein (ARFGAP1) involved in membrane trafficking was up-regulated while vacuolar protein sorting-associated protein 4B (VPS4P) and 4 members of Ras-related proteins (RAB5A, RAB5B, RAB7A and RAB11A) were down-regulated (Supplementary File S1).

These data run parallel to increased phospholipid content and demonstrate that Prdx6 is involved in membrane arrangement and vesicle trafficking, but it remains to be determined which activity of Prdx6, either the peroxidase, the PLA2, the LPAT, or a combination of these, is responsible for this role.

#### 3.4.2. Redox Proteome

Down-regulation of Prdx6 should be expected to alter the redox homeostasis by diminution of its antioxidant capacity, so we analyzed the Redox Proteome, namely, the set of proteins undergoing redox changes at sensitive cysteines on siRNA-Prdx6 treatment in HepG2 cells. A large set of nearly one thousand cysteinyl peptides were detected (Supplementary File S2). We selected those differential Cys-peptides with statistical significance (*p* < 0.05) and >1.5-fold or <0.6-fold change in their Cys reduced/oxidized ratio. The main changes were detected in enzymes of carbon and glutathione metabolism, pentose phosphate pathway, citrate cycle, fatty acid metabolism, biosynthesis of aminoacids, and Glycolysis/Gluconeogenesis.

A significant redox target was Hexokinase-2 (HK2) at Cys368, that was half reduced in HepG2 cells but was reversibly oxidized after siRNA-Prdx6 treatment (Table 1). HK2 is known to play a critical role in the connection between metabolic and cell survival pathways and is upregulated in hepatocellular carcinoma and other tumors [46,47]. HK2 associates with the outer mitochondrial membrane via a binding motif at the N-terminal that interacts with voltage dependent anion channel (VDAC).

**Figure 3:**
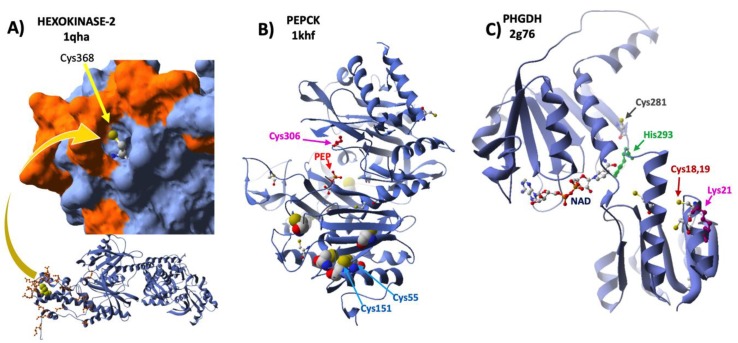
Structural mapping of redox sensitive Cys. The 3D structures of three enzymes of carbohydrate metabolism, HK2, PCK, and PHGDH, with the indicated PDB ID codes, were analyzed to map the Cys residues that showed significant redox changes upon Prdx6 silencing in HepG2 cells. (**A**) One subunit of HK2 is shown below in a ribbon model with Cys368 atoms as yellow Van der Waals spheres and sidechains of residues involved in interaction contacts with VDAC1 highlighted in orange; the C-terminus is enlarged in molecular surface mode, except for Cys368, with the same highlighting criterium as below. (**B**) PCK in ribbon mode and detected Cys residues with atoms as Van der Waals spheres, color CPK; one molecule of substrate PEP (red) is bound at the active site; the positions of Cys306, Cys55 and Cys151 are indicated with arrows. (**C**) PHGDH in ribbon cartoon with the coenzyme NAD bound at the active site cleft; sidechains of redox modified Cys residues upon Prdx6 silencing are shown in CPK color; acetylation and sumoylation sensitive Lys21 is shown in purple next to redox sensitive Cys18-Cys19; catalytic His283 is shown in green with the redox sensitive Cys281 in the neighborhood.

This association provides a metabolic advantage by bringing HK2 closer to the ATP production site, and an apoptosis suppressive capacity by competing with proapoptotic factors for the binding to VDAC [48]. Cys368 occupies a central position in the VDAC-HK contact area [49] (Figure 3A) and it is conceivable that its oxidative modification on siRNA-Prdx6 treatment could have altered its interaction with VDAC, affecting its subcellular localization and inducing a decrease in the rate of glucose phosphorylation and entry into the cell.

Prdx6 was abundant enough to be clearly detected (24 peptides) even in silenced cells (Supplemetary Files S1 and S2). The peptide containing the “non-catalytic” Cys91 was prominent and showed sensitivity to reversible oxidation, as it was fairly reduced in HepG2cells but was significantly more oxidized after siRNA-Prdx6 treatment (Table 1). The peptide containing its catalytic peroxidatic Cys47 did not show significant reversible redox changes (See data in Supplementary File S2). The “extra” Cys91 is not conserved among members of the 1-Cys subfamily of Prx and has been thoroughly neglected or routinely substituted by Ser in the recombinant protein and other experimental setups. However, we have found that Cys91 of human Prdx6 was specifically sensitive to reversible oxidation in glutaredoxin 1 (Grx1) down-regulated HepG2 cells and had shown that it is prone to glutathionylation in recombinant Prdx6 [28]. Experiments are under way to elucidate whether this “extra” Cys in human Prdx6 is relevant to its structure and function in the context of redox signaling.

Silencing of Prdx6 in HepG2 cells led to redox changes in Cys191 of the intracellular chloride ion channel-1 (CLIC1). This is a soluble globular protein that can undergo an extended conformational change to get embedded in intracellular membranes, mainly in the nucleus, and form a Cl- ion channel that has been linked to apoptosis, pH, cell volume and cell cycle regulation [50,51]. The soluble form of CLIC1 consist of two domains, the N-terminal one with a thioredoxin fold similar to glutaredoxin and the C-terminal typical of the GST superfamily; it also contains a glutathione binding site [52]. These properties of CLIC1 suggested that it may be under some kind of redox control. Cys191, which is located in the GST domain of CLIC1, was more reduced in siRNA-Prdx6 treated cells (See data in Supplementary File S2). Coincidentally, Prdx6 interacts with GST-π during the peroxidase catalytic cycle [53]. It is tempting to hypothesize on a possible redox relationship between Prdx6 and CLIC1 contributing to the effects of Prdx6 silencing on cell swelling and cell cycle arrest described above. 

Redox changes in two enzymes of the Glycolysis/gluconeogenesis pathway are worth of mention. Eight cysteines were detected out of a total of 12 in the sequence of mitochondrial PEP-carboxykinase (PCK2), which is composed of two large domains, with the active site in between, that move relative to each other during the catalytic cycle. Of these Cys, seven are clustered in the C-terminal domain (Figure 3B), but only the peptide containing Cys55 and Cys63, conserved in eukaryotes, showed significant redox changes. Cys55 could form a disulfide bond with Cys151, which would be ≈3Å apart. The enzyme is inactivated by glutathionylation of Cys306, which is close to the active site, in a way reversed by SH2 [54]. We have detected this cysteine in all the samples in a highly reduced state (red/ox > 20) insensitive to Prdx6 silencing, allowing for full activity (Supplementary File S2).

3-phosphoglycerate dehydrogenase (PHGDH) plays a critical role in a relevant metabolic diversion of Glycolysis that leads to serine, a precursor of proteins, phospholipid head groups and glutathione and driving force for the folate and methionine cycles [55]. Its gene is overexpressed in several types of cancers, where it can catalyze the reduction of 2-oxo-glutarate to 2-hydroxyglutarate, a potent oncometabolite [56]. In the current study, we have found several redox sensitive Cys in PHDH all of which are in a highly reduced state (red/ox ratios ≈ 5–30). However, Cys281 and the peptide containing contiguous Cys18Cys19, three Cys conserved in mammals, were significantly less reduced under conditions of Prdx6 silencing in HepG2 cells (Table 1 and Supplementary File S2). Cys281, which was identified as a target for S-nitrosation in a high-throughput study [57], is in the NAD-binding catalytic domain, close to the essential active site residue His293, while Cys18Cys19 are located in the so called allosteric regulatory domain in close proximity to Lys21 (Figure 3C), which is a target for acetylation and sumoylation [58]. Reversible redox changes in these sensitive Cys of the enzyme could affect its catalytic and regulatory properties [59].

## 4. Conclusions

The complex wide effects of moderate Prdx6 down-regulation should be a reflection of its moonlighting properties acting as peroxidase, phospholipase, and LPC-acyltransferase. The response of HepG2 cells to Prdx6 silencing spans from metabolic and signaling pathways remodeling to alterations in the redox proteome and membrane turnover with effects in cell cycle progression and proliferation. The role of Prdx6 in cancer cells has been studied previously with conflicting outcomes. Most reports point to a pro-proliferating and antiapoptotic action of Prdx6, but some studies have found the opposite effect, depending on the stage of tumor development. Moreover, these effects have not always been assigned to a particular activity of Prdx6, either peroxidase, PLA2, or LPCAT, neither a common mechanism has been provided.

In the study reported herein Prdx6 silencing down to ≈40% would represent a situation mimicking a cellular response to endogenous or exogenous stimuli under normal or oxidative stress conditions.

It appears that the cell responds to Prdx6 down-regulation by initiating a biosynthetic program, leading to membrane and vesicle trafficking rearrangement with signs of entering a cell cycle G1 phase but an inability to proceed on to the S phase. The mechanisms behind these phenomena involve reversible thiol oxidative changes in key proteins. HK2-Cys368 and Prdx6 “extra” Cys91 stand out as relevant thiol switches. The former could affect association with the mitochondrial membrane with consequences on the rate of glucose catabolism and mitochondrial permeability; the latter is not conserved among the 1-Cys type Peroxiredoxins and has been given little attention so far, but could turn out to bear relevant functions in human cells.

## Figures and Tables

**Table 1:** Reversible thiol redox changes in specific sensitive Cys.

	siRNA Prdx6	HK2-Cys^368^	Prdx6-Cys^91^	PHGDH-Cys^18-19^
**Ratio red/ox ± sd (** ***n*** **= 4)**	−	**0.58** ± 0.04	**1.78** ± 0.33	**10.43** ± 2.75
	**+**	**0.26** ± 0.03	**0.92** ± 0.06	**4.98** ± 1.98
**Fold Change**		**0.45**	**0.52**	**0.48**
*p*-value		1.6 × 10^−5^	2.2 × 10^−2^	2 × 10^−4^

The reduced/oxidized ratio of specific cysteines in three proteins, specifically discussed in the main text, suffering reversible redox changes upon Prdx6 silencing as determined by the quantitative redox proteome protocol described in Materials and Methods. The ratio values (± s.d.; *n* = 4) are shown and the fold change provoked by Prdx6 silencing is also indicated together with the statistical significance index (*p*-value) for HepG2 cells.

## Supplementary Materials

The following are available online at https://www.mdpi.com/2076-3921/8/11/505/s1; Supplementary File S1: Excel file showing results of global quantitative proteomic analysis in Prdx6 silenced HepG2 cells. The first sheet shows protein ID, protein name, gene name and number of unique peptides in the first columns. NT and siRNA-Prdx6 indicate treatment with unspecific “non-target” siRNA and with specific siRNA, respectively. Columns M and N with blue colored headings show the calculated fold change induced by siRNA-Prdx6 treatment together with the statistical significance index (*p* value). Statistically significant data have been selected and disclosed into the second sheet with the fold change colored green and red for down- and up-regulated values, respectively. The sheets named “GOTERM_Enrichment” and “KEGG_Enrichment” show the results of “Biological Process GO-term” and “KEGG pathway” enrichment analysis of the set of differentially expressed proteins on siRNA-Prdx6 treatment; Supplementary File S2: Excel file showing results of cysteinyl peptides proteomic quantitative analysis of in Prdx6 silenced HepG2 cells. The peptide sequence, accession ID and protein name are shown in the first columns. NT and siRNA-Prdx6 indicate treatment with unspecific “non-target” siRNA and with specific siRNA, respectively. The data in columns D to K are the reduced/oxidized ratio values for each of 4 replicas for every Cys-peptide; NA, data not available. Columns L and M, colored orange show the fold change of the red/ox ratio induced by siRNA-Prdx6 treatment together with the statistical *p* value for significance. The second sheet with the fold change colored green and red for down- and up-regulated values, respectively shows the selected significant fold change values (column E) induced by Prdx6 silencing ordered by increasing *p*-values (column F); the position of the Cys residue in the sequence of the protein is given in column D and a horizontal double red line marks the statistical threshold for significance; Supplementary File S3: Excel file showing results of metabolomic analysis in Prdx6 silenced HepG2 cells. Columns A and B show the metabolite family and the name of the metabolite, respectively. The data show the normalized quantitative values for each metabolite in each of 4 replicas for the treatment with unspecific siRNA (NT) and siRNA-Prdx6. NA, missing or outlier value. The fold change induced by Prdx6 silencing is shown in column K, together with the statistical *p*-value for significance, with values <0.05 highlighted in yellow.
